# Effects of daily bathing with 2% chlorhexidine compared to bathing with soap and water on risk of death, clinical severity, and intensive care unit length of stay in critically ill patients: an updated systematic review and meta-analysis

**DOI:** 10.1007/s10096-026-05513-5

**Published:** 2026-04-18

**Authors:** Edison Vitório de Souza Júnior, Claudiomiro da Silva Alonso, Camila Cláudia Campos, Diego Pires Cruz

**Affiliations:** 1 School of Nursing, Department of Basic Nursing, Federal University of Minas Gerais, Belo Horizonte, Minas Gerais Brazil; 2 Department of Health, State University of Southwest Bahia, Jequié, Bahia Brazil

**Keywords:** Nursing, Intensive care units, Critical care nursing, Quality of health care, Hospital administration

## Abstract

**Objective:**

To evaluate the effectiveness of daily bathing with 2% CHG compared with traditional SAW bathing on risk of death, clinical severity, and ICU length of stay in critically ill patients.

**Methods:**

Between November 10 and 30, 2025 two reviewers independently conducted literature searches in six databases (PubMed, Cochrane, Embase, Scopus, ScienceDirect, and Web of Science). We included studies published in indexed journals that met the predefined PICOTT criteria. We calculated Risk Ratio, Mean Difference and Standardized Mean Difference with 95% confidence intervals for all analysis using random-effects models.

**Results:**

Ten studies were included, comprising a total of 33,860 patients. Of these, 18,102 (53.5%) received traditional bathing with SAW, while 15,758 (46.5%) received baths with 2% CHG. Bathing with 2% CHG did not reduce the risk of death (RR 0.93 [95% CI 0.86; 1.00], *p* = 0.05, I²=4%) and did not reduce clinical severity scores (SMD − 0.09 [95%CI − 0.33; 0.14]; *p* = 0.43; I² = 95%), with both outcomes supported by very low certainty of evidence. Patients receiving 2% CHG bathing had a mean ICU length of stay 0.07 days longer than those receiving SAW in the ICU (MD 0.07 [95% CI 0.04, 0.10], *p* < 0.001, I2 = 0%) However, the certainty of evidence was low and the magnitude of this effect is unlikely to be clinically meaningful.

**Conclusion:**

Daily bathing with 2% CHG was not associated with a statistically significant reduction in risk of death or clinical severity. A small increase in ICU length of stay (0.07 days) was observed.

**Supplementary Information:**

The online version contains supplementary material available at 10.1007/s10096-026-05513-5.

## Introduction

Daily bathing with 2% chlorhexidine gluconate (2% CHG) has been extensively investigated [1–6]. This healthcare practice involves the application of a broad-spectrum antiseptic agent with well-established antimicrobial activity, capable of exerting either bactericidal or bacteriostatic effects depending on its concentration [7, 8].

Studies have reported conflicting findings regarding the benefits of 2% CHG. Most investigations have focused on infectious outcomes—particularly the prevention of bloodstream infections associated with invasive devices [1, 3, 4, 9]. However, other studies have failed to demonstrate consistent benefits [10–12], highlighting ongoing uncertainty about the true magnitude and clinical relevance of these effects.

Although prior studies have primarily focused on infectious outcomes, the clinical relevance of daily 2% CHG bathing extends beyond microbiological endpoints. In critically ill patients, healthcare-associated infections are consistently associated with increased organ dysfunction, prolonged ICU stay, and higher mortality [13]. Therefore, if CHG bathing effectively reduces infectious burden, a downstream impact on global severity indices, length of stay, or survival could be biologically plausible. However, the extent to which infection prevention strategies translate into improvements in these broader clinical outcomes remains unclear, particularly in analyses restricted to homogeneous bathing protocols. Clarifying this relationship is essential to determine whether potential reductions in infection meaningfully alter patient trajectory.

The most recent meta-analysis on this topic included 20,188 patients admitted to intensive care units (ICUs) [14]. The authors found no statistically significant association between the use of 2% CHG and either mortality risk or ICU length of stay.

However, this analysis incorporated studies with substantial heterogeneity in both intervention and control protocols, including variations such as daily versus alternate-day bathing and the use of comparators other than the conventional soap-and-water bath (SAW). Such methodological inconsistencies may have compromised cross-study comparability and reduced the ability to detect potential effects on broader clinical outcomes.

Thus, despite the recognized relevance of this topic to nursing and healthcare practice, there remains a need for a comprehensive analysis of studies adopting more homogeneous intervention and control protocols. From this perspective, our objective was to evaluate the effectiveness of daily bathing with 2% CHG, compared with traditional soap-and-water (SAW) bathing, on risk of death, clinical severity, and ICU length of stay in critically ill patients.

## Methods

### Study design

This systematic review and meta-analysis was conducted in accordance with Cochrane guidelines for developing systematic reviews and meta-analyses [15, 16].

The data presented here are derived from additional outcomes of the primary study designed to compare daily bathing with 2% chlorhexidine versus traditional bathing with soap and water in reducing infections in critically ill patients. The protocol for this research was registered in advance in the International Prospective Register of Systematic Reviews (PROSPERO) with the registration number CRD420251174669.

During the preparation of this work, the authors used ChatGPT to refine sentences previously created by the authors in order to ensure better comprehension of the English language. After using this tool/service, the authors reviewed and edited the content as needed and assume full responsibility for the content of the published article

### Eligibility criteria

We included studies published in indexed scientific journals that fully addressed the PICOTT framework:

- (P) – critically ill adult patients;
- (I) – daily bathing with 2% CHG, regardless of formulation, including liquid solutions, chlorhexidine-impregnated cloths or wipes, and chlorhexidine-containing soap formulations;
- (C) – traditional bathing with SAW;
- (O) – risk of death, clinical severity and average length of stay in the ICU;
- (T) – randomized and observational studies;
- (T) – no temporal restrictions were applied, and studies were eligible regardless of geographic location or language.

Studies were excluded if they lacked a control group, included overlapping populations, administered the intervention more than once daily, or used chlorhexidine concentrations other than 2%. We also excluded studies in which the control group received alternatives to SAW, as well as those in which chlorhexidine was combined with additional co-interventions in the experimental group. Variations in intervention protocols and contextual characteristics across studies are summarized in Supplementary Material.

### Search strategy and data extraction

Between November 10 and 30, 2025, two reviewers independently conducted the literature searches in six databases. The main search terms used were: “critically ill”, “critical illness”, “intensive care units”, “intensive care”, “baths”, “chlorhexidine”, “hospital infection”, “bloodstream infections”, “infections”, “death”, “mortality”, “end of life”, “severity of illness index”, “clinical severity”, “stay”, “length of stay”, and “LOS”. The complete search strategy is described in the supplementary material.

To ensure the comprehensiveness of the search strategy, we conducted an additional verification step by screening the reference lists of previous systematic reviews addressing chlorhexidine bathing in critically ill patients. All primary studies included in these reviews were cross-checked against the studies identified in our search strategy. This procedure allowed us to verify the overlap between previously published evidence syntheses and the studies retrieved in the present review, minimizing the risk of omission of eligible studies. No additional eligible studies were identified beyond those already captured through the database searches.

All records retrieved from the databases were imported into the Mendeley reference management software, where duplicate records were identified and removed before the screening process. After duplicate removal, the remaining records were screened by two independent reviewers.

Data extraction was conducted using a standardized form. The extracted information included study design, country, sample characteristics, intervention and control protocols, and outcomes of interest. After data collection, the information extracted from the articles was organized in a structured spreadsheet to categorize the main results.

### Outcomes and subgroup analyses

The primary outcomes were the risk of death, clinical severity, and mean length of ICU stay. For the outcome risk of death, pooled analyses were conducted using the total number of events reported in each group, without conversion into mortality rates. ICU length of stay was analyzed as a continuous variable and expressed as the mean difference in days between groups.

Clinical severity, in this context, refers to the degree of acute physiological derangement and predicted mortality risk, as quantified by validated ICU prognostic scoring systems, particularly the APACHE model [17]. These composite indices integrate multiple physiological variables and chronic health conditions to generate an overall estimate of illness severity. Because they capture systemic physiological burden rather than isolated infectious events, severity scores enable the assessment of whether infection-prevention strategies may influence the broader clinical trajectory beyond discrete outcomes.

Subgroup analyses were conducted a posteriori to explore potential sources of heterogeneity. The following subgroup was considered:

1. type of ICU: medical and mixed ICUs. Information regarding ICU type was extracted directly from the included studies. A medical ICU (MICU) was defined as a unit that admitted only patients with medical (non-surgical) conditions. Patients are typically admitted due to organ dysfunction or failure resulting from acute illnesses or acute exacerbations of chronic diseases. Mixed ICUs were defined as units that admitted patients from multiple specialties, such as medical, neurosurgery, clinical/surgery, trauma and non-trauma emergency surgery.
2. Type of instruments for assessing clinical severity: Acute Physiology and Chronic Health Evaluation (APACHE), versions II and III.

### Quality assessment

All stages of the review process, including title and abstract screening, full-text assessment, data extraction, risk of bias evaluation, and certainty of evidence assessment, were performed independently by two reviewers. Any disagreements regarding study eligibility, extracted data, or methodological assessments were resolved through discussion between the two reviewers until consensus was reached. During this process, decisions were guided by the predefined eligibility criteria structured according to the PICOTT strategy and by the formal recommendations of the risk-of-bias assessment tools.

The risk of bias of the included studies was assessed using the Risk Of Bias In Non-randomized Studies of Interventions (ROBINS-I) tool for non-randomized studies and the Cochrane Risk of Bias 2 (RoB 2) tool for randomized trials. Publication bias analysis was not performed using the funnel plot because we did not reach the minimum number of ten studies required for the analysis [16]. We used the Grading of Recommendations Assessment, Development, and Evaluation (GRADE) tool to assess the certainty of the evidence. All of these quality assessments are available in supplementary material.

### Statistical analysis

All analyses were conducted in accordance with the Cochrane Handbook for Systematic Reviews of Interventions (version 6.5) [16], using RevMan (Cochrane Centre, The Cochrane Collaboration, Denmark).

The random effects model used was Restricted Maximum Likelihood (REML). We calculated Risk Ratios (RR), Mean Difference (MD) and Standardized Mean Difference (SMD) with 95% confidence intervals (CI) for all analyses.

Statistical heterogeneity was assessed using the I² statistic. Values of 25% or higher were considered indicative of substantial heterogeneity and therefore warranted further investigation [18]. To verify the consistency of the findings, sensitivity analyses were performed by sequentially removing each study (leave-one-out method), allowing us to observe whether any of them had a disproportionate influence on the estimated overall effect.

Studies that provided data only in medians and interquartile ranges had their values converted to mean and standard deviation after detecting the absence of asymmetry [19, 20]. This strategy was adopted in order to standardize the analyses as required by Review Manager (RevMan) (Cochrane Center, The Cochrane Collaboration, Denmark).

For cluster-randomized trials (CRTs) that reported only raw data without adjustment for clustering, the effective sample size approach recommended by the Cochrane Handbook was applied. The design effect was calculated as 1 + (𝑚 − 1) × 𝐼CC, assuming an intracluster correlation coefficient (ICC) of 0.01 [21]. For dichotomous outcomes, both the number of participants and events were divided by the design effect to avoid unit-of-analysis errors. For continuous outcomes, only the number of participants was adjusted using the design effect, while the means and standard deviations were kept unchanged [16].

The results of the meta-analysis were presented visually using forest plots with effect estimates and confidence intervals. Analyses of risk of bias and certainty of evidence, as well as the characteristics of the included studies, were presented in tables.

## Results

**Fig. 1:**
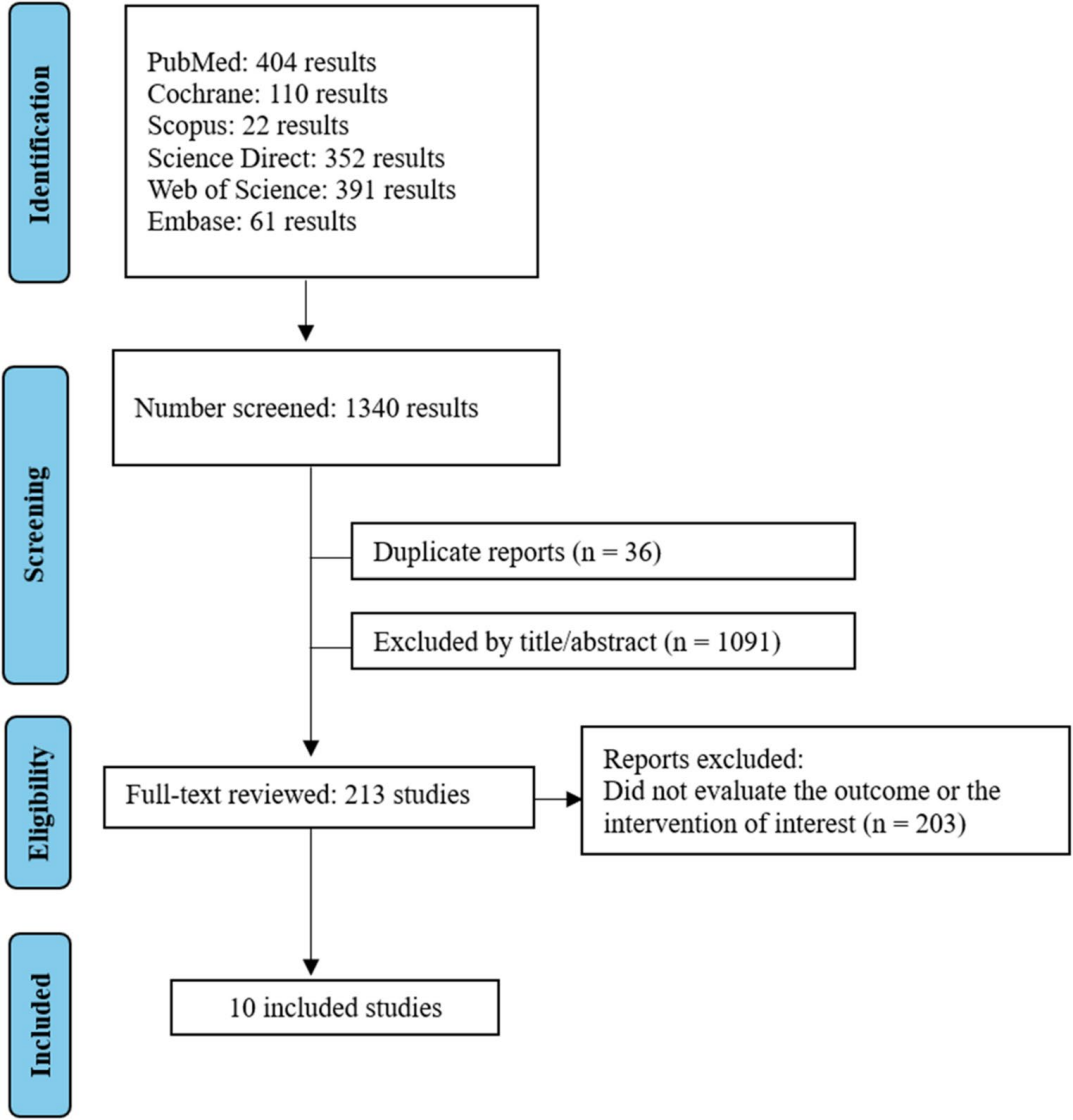
PRISMA flowchart for study screening and selection. *PRISMA* preferred reporting items for systematic reviews and meta-analyses

Figure 1 demonstrates the systematic approach to study selection. After a rigorous process conducted by two independent authors, ten studies that fully met the inclusion criteria were included.

Of the 10 studies included, three were cluster randomized trials (CRT), and seven were non-randomized controlled trials (non-RCTs), comprising a total of 33,860 patients. Of these, 18,102 (53.5%) received traditional bathing with SAW, while 15,758 (46.5%) received baths with 2% CHG. Study characteristics are reported in Table 1.

**Table 1:** Baseline characteristics of included studies

Primary author (year)	Journal	Design	Country	Participants SAW/CHG	Male (%) SAW/CHG	Aged SAW/CHG
Bleasdale (2007) [3]	Archives of internal medicine	CRT	United States	445/391	59.8/59.8	52 ± 15^a^/53 ± 16^a^
Cassir (2015) [22]	American Journal of Infection Control	Non-RCT	France	175/150	58.2/61.3	61(48–73)^b^/58(46–68)^b^
Chang (2025) [23]	Microorganisms	Non-RCT	Taiwan	1330/1372	58.2/52.8	69.7 ± 14.9^a^/68.8 ± 24.7^a^
Duszyńska (2017) [9]	Anaesthesiology Intensive Therapy	Non-RCT	Poland	92/105	65,2/57,1	64 ± 18^a^/62 ± 18^a^
Kengen (2018) [10]	Critical Care and Resuscitation	Non-RCT	Australia	3364/2970	61,6/62,1	61.1 ± 17.7^a^/62.6 ± 17.3^a^
Lin (2025) [11]	Nursing in Critical Care	Non-RCT	Taiwan	4098/1384	62.6/61.3	67.2 ± 15.4^a^/66.4 ± 15.4^a^
Reis (2022) [12]	The Brazilian Journal of infectious diseases	CRT	Brazil	620/867	49.3/47	63.7^a^/58.3^a^
Suh (2021) [4]	Antimicrobial Resistance and Infection Control	Non-RCT	South Korea	259/242	59.8/57.9	74(62–81)^b^/73(57–81)^b^
Tarakçıoğlu Çelik (2025) [1]	Mikrobiyoloji Bülteni	Non-RCT	Turkey	31/30	67.7/53.3	63.6 ± 16.8^a^/62.6 ± 12.4^a^
Tomazini (2026) [24]	The Lancet Regional Health - Americas	CRT	Brazil	7688/8247	52.5/52.3	64.2 ± 17·4/64.4 ± 17.3a

*a* average value, *b* median (interquartile range), *CHG* 2% chlorhexidine gluconate, *CRT* cluster randomized trial, *SAW* soap and water

**Fig. 2:**
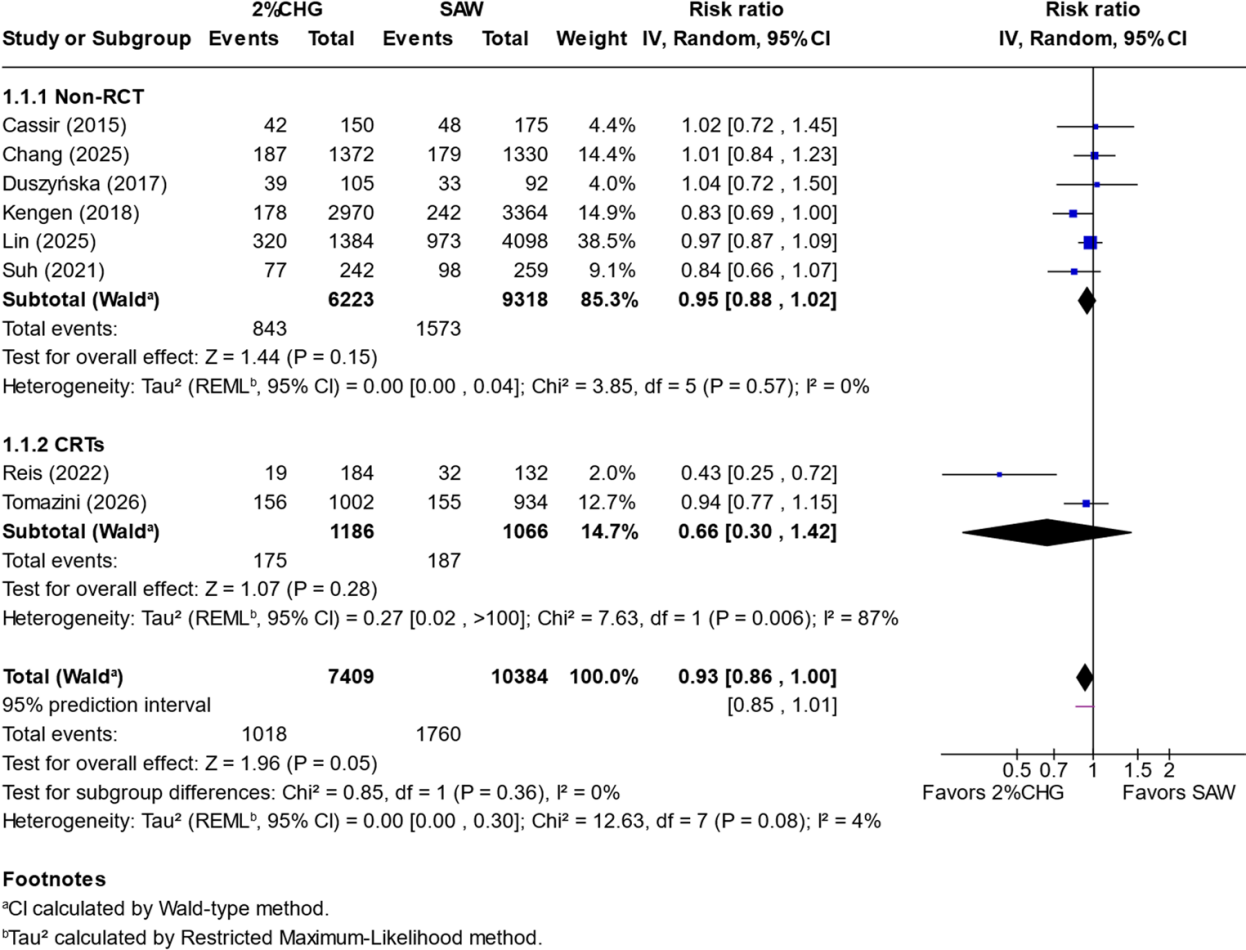
Deaths according to the study design. *2%CHG* 2% gluconate chlorhexidine, *SAW* soap and water, *Non-RCT* non-randomized controlled trial, *CRTs* clusters randomized trials

Risk of death was assessed in eight studies, totaling 17,793 patients. Of these, 7,409 (41.6%) received bathing with 2% CHG (Fig. 2). A lower risk of death was observed in the 2% CHG group compared with soap and water, with a borderline effect (RR 0.93 [95% CI 0.86; 1.00], *p* = 0.05, I²=4%) (certainty of evidence: very low). Statistical heterogeneity was low, indicating high consistency across studies. The 95% prediction interval ranged from 0.85 to 1.01, suggesting that future studies may show either a small benefit or no effect of the intervention on the number of deaths (Fig. 2).

The leave-one-out sensitivity analysis indicated that the study conducted by Reis et al. (2022) [12] contributed substantially to the high heterogeneity observed among the CRTs (I² = 87%). After removing this study from the analysis, heterogeneity was reduced to I² = 0%. However, the pooled estimate remained non-significant (RR 0.94 [95% CI 0.88; 1.01], *p* = 0.12, I2 = 0%).

**Fig. 3:**
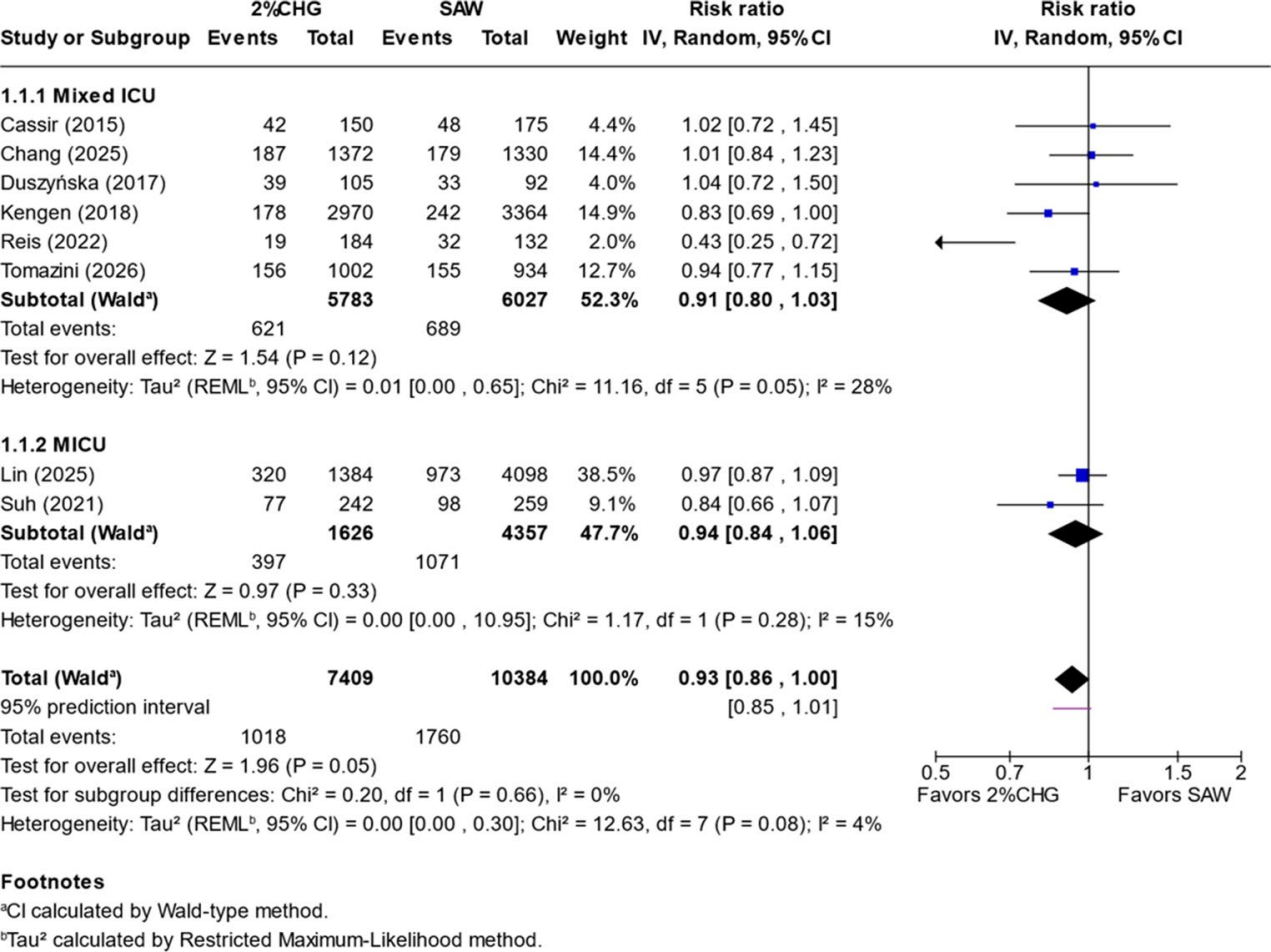
Deaths according to the ICU type. *2%CHG* 2% gluconate chlorhexidine, *SAW* soap and water, *ICU* intensive care unit, *MICU* medical intensive care unit

In the subgroup analysis by ICU type, data were stratified into two categories: medical and mixed ICUs (Fig. 3). It is observed that there was also no statistically significant difference between the units (*p* = 0.66) and the overall statistical significance remained borderline (*p* = 0.05).

**Fig. 4:**
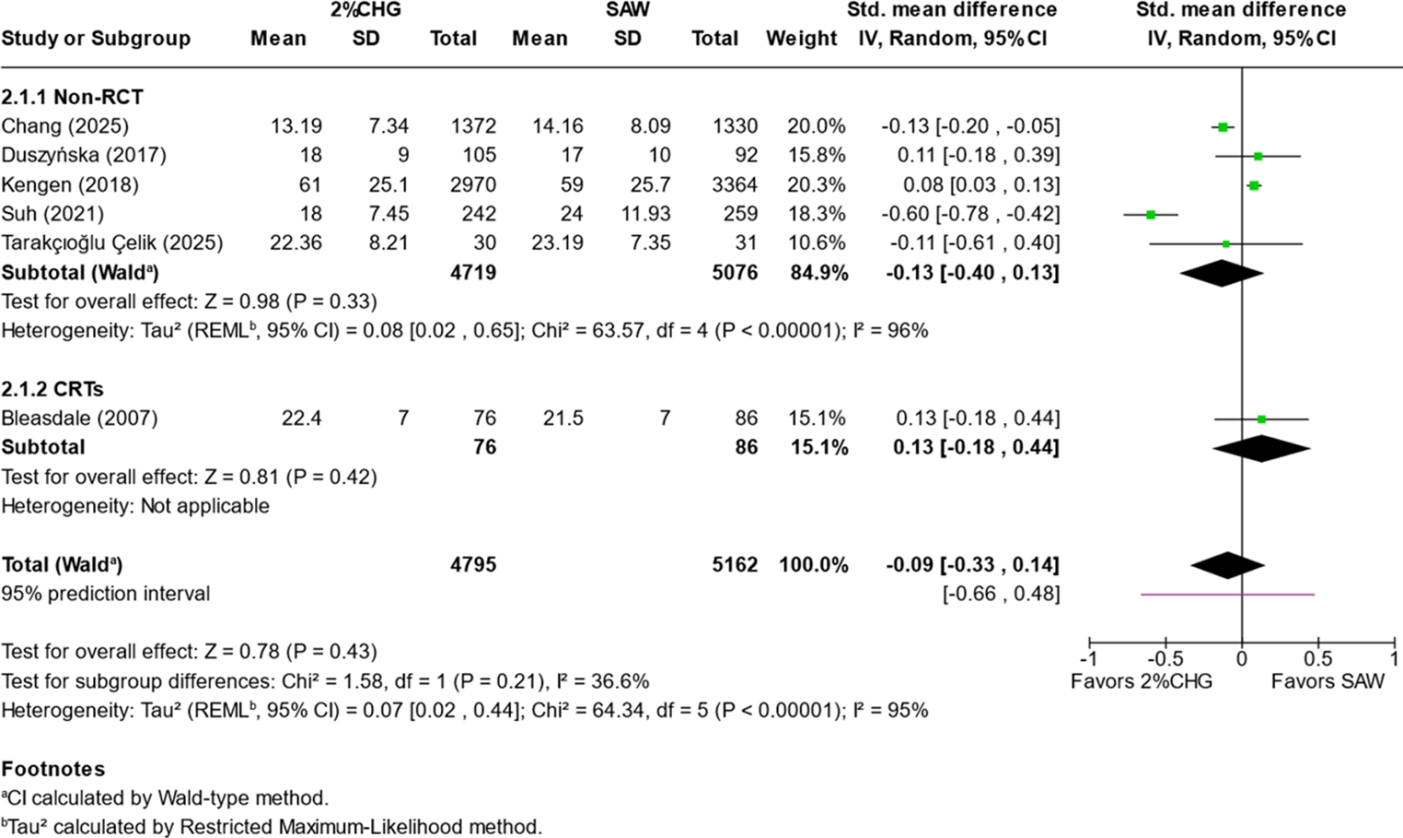
Clinical severity assessed using the APACHE score. *2% CHG* 2% chlorhexidine gluconate, *SAW* soap and water, *APACHE,* acute physiology and chronic health evaluation

Clinical severity was assessed in six studies, totaling 9,957 patients. Of these, 4,795 (48.2%) received a bath with 2% CHG (Fig. 4). The pooled analysis of APACHE scores showed no statistically significant difference between groups (SMD − 0.09 [95%CI − 0.33; 0.14]; *p* = 0.43; I² = 95%) (certainty of evidence: very low). However, heterogeneity was extremely high, indicating substantial variability across studies.

In the leave-one-out analysis, exclusion of the study by Suh et al. (2021) [4]. reduced heterogeneity from I² = 95% to I² = 75%, although it remained considerable and the result was not statistically significant (SMD 0.01 [95%CI − 0.12; 0.14]; *p* = 0.88; I² = 75%).

**Fig. 5:**
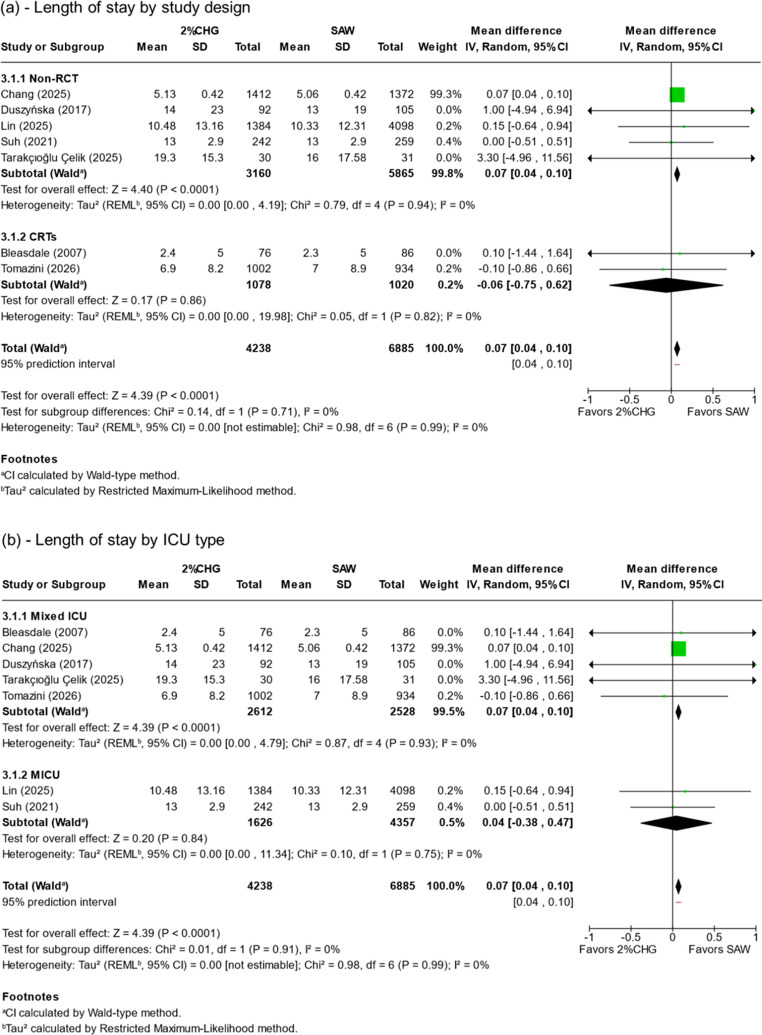
ICU length of stay according to study design and ICU type. *2%CHG* 2% gluconate chlorhexidine, *SAW* soap and water, *ICU* intensive care unit, *MICU* medical intensive care unit, *CRT* cluster-randomized trial, *Non-RCT* non-randomized controlled trial

The length of stay in the ICU was evaluated in seven studies, totaling 11,123 patients (Fig. 5). Of these, 4,238 (38.1%) received a bath with 2%CHG (Fig. 4).

The meta-analysis showed a statistically significant difference in ICU length of stay between patients receiving daily bathing with 2% chlorhexidine and those receiving soap-and-water bathing (Fig. 4). However, the magnitude of the effect was minimal (MD = 0.07 days), corresponding to approximately 1.7 h, which is unlikely to be clinically meaningful (MD 0.07 [95%CI 0.04; 0.10], *p* < 0.001, I^2^ = 0%) (certainty of evidence: low). Furthermore, the pooled estimate was overwhelmingly driven by a single large study contributing more than 99% of the weight. Randomized cluster trials did not demonstrate a significant effect, suggesting that the overall finding should be interpreted cautiously.

## Discussion

Some studies have already reported that bathing with 2% CHG is associated with a reduction in infections in critically ill patients [3, 4, 9, 22]. Based on these findings, it was hypothesized that such effects could also extend to reduction in the risk of death and clinical severity; however, this hypothesis was not consistently confirmed in the present analysis.

Although the pooled estimate suggested a borderline reduction in mortality, this finding should be interpreted with caution given the structure and limitations of the available evidence. According to the GRADE approach, the certainty of evidence for mortality was rated as very low, indicating that the true effect may be substantially different from the observed estimate. Mortality in critically ill patients is a distal and multifactorial outcome, predominantly determined by baseline organ dysfunction, comorbid burden, and therapeutic intensity rather than by isolated preventive interventions [25, 26].

This finding is consistent with previous evidence syntheses. The most recent meta-analysis by Peixoto et al. (2024) [14], which primarily focused on infectious outcomes, also did not demonstrate a statistically significant reduction in mortality associated with chlorhexidine bathing. Similarly, large cluster-randomized trials evaluating decolonization strategies, such as those reported by Bleasdale et al. [3] and Tomazini et al. [24], although demonstrating reductions in certain healthcare-associated infections, did not report a consistent mortality benefit. These converging findings reinforce the notion that reductions in colonization or bloodstream infections do not necessarily translate into measurable survival advantages in heterogeneous ICU populations.

In addition, to aligning with prior evidence, the present review incorporates recently published large-scale studies and restricts inclusion to daily 2% chlorhexidine bathing compared exclusively with soap and water, thereby reducing the intervention heterogeneity observed in previous reviews.

Taken together, this pattern of evidence highlights not only the biological and clinical complexity underlying mortality outcomes, but also the methodological challenges inherent in isolating a causal effect of chlorhexidine bathing. In this context, the predominance of non-randomized studies substantially limits causal inference. Given that mortality and severity scores are strongly influenced by baseline comorbidities, underlying physiological derangement, and institutional care practices, such designs are particularly susceptible to residual confounding. Even when statistical adjustments are applied, imbalance in baseline risk cannot be fully excluded.

In addition, the fragility of the estimate demonstrated by sensitivity analyses in which heterogeneity changed markedly after removal of individual studies further reduces confidence in the stability of the observed effect. These limitations contributed to downgrading the certainty of evidence due to risk of bias, indirectness, and imprecision.

Part of this instability may be explained not only by methodological limitations, but also by clinical heterogeneity across the included settings. Reis et al. (2022) [12], for instance, included patients from clinical, neurosurgical, traumatic and non-traumatic emergency ICUs, as well as a mixed ICU involving both clinical and surgical patients. Such variability in ICU profiles and consequently in patient characteristics, severity patterns, and care trajectories likely contributed to inconsistent effect estimates, thereby amplifying statistical heterogeneity within the meta-analysis.

In addition, to clinical variability, methodological quality may also have influenced the stability of the pooled estimates. Therefore, to disentangle the potential contribution of risk of bias from that of clinical heterogeneity, a sensitivity analysis excluding studies classified as having a high risk of bias was conducted (Supplementary Material, Fig. 4). After excluding these studies, the results for number of deaths remained consistent with the main analysis, showing no statistically significant difference between groups (RR 0.89 [95%CI 0.78; 1.02], *p* = 0.09; I² = 47%).

This finding suggests that the inclusion of studies with higher risk of bias did not substantially alter the overall interpretation of the results. Nevertheless, the persistent imprecision of the confidence interval and the remaining heterogeneity further justify the classification of the certainty of evidence as very low. Taken together, the current evidence does not provide sufficiently robust grounds to support a mortality benefit.

While the mortality analysis appeared methodologically stable after adjustment for risk of bias, a different pattern emerged when examining clinical severity outcomes. The interpretation of clinical severity outcomes is particularly constrained by extreme statistical heterogeneity and conceptual variability across studies. Notably, this heterogeneity persisted despite the use of the same severity assessment tool across the included studies. Although the instrument itself was consistent, differences in timing of assessment, baseline case-mix, ICU profile, and underlying patient trajectories likely contributed to substantial variability in reported scores.

Unlike mortality, severity scores are dynamic indices influenced by baseline physiological status and the moment of measurement, which reflects the quality of the data [27]. Therefore, pooling these heterogeneous measures assumes a level of comparability that may not fully exist. The dramatic reduction in heterogeneity observed after exclusion of certain studies suggests that the pooled estimate is highly sensitive to study-level characteristics rather than reflecting a consistent intervention effect.

Additionally, the biological plausibility of an antiseptic bathing protocol substantially modifying global severity indices is limited. Thus, the absence of a clear effect should be interpreted in light of measurement variability and conceptual dispersion rather than as definitive evidence of inefficacy.

A sensitivity analysis excluding studies classified as having a high risk of bias was conducted (Supplementary Material, Fig. 5). For clinical severity, the sensitivity analysis showed a small but statistically significant difference (SMD 0.08 [95% CI 0.04; 0.13], *p* < 0.001, I^2^ = 0%) with no observed heterogeneity. However, this result was largely driven by a single large study, which accounted for most of the statistical weight in the analysis. The dependence of the pooled estimate on one influential study further contributed to downgrading the certainty of evidence due to concerns about imprecision and potential small-study effects. Accordingly, the available data do not allow a reliable inference regarding an effect on global severity indices.

Unlike mortality, clinical severity scores were not formally synthesized in previous systematic reviews on chlorhexidine bathing, leaving uncertainty regarding potential effects on intermediate physiological indicators. By incorporating validated severity instruments such as APACHE scores, the present review expands the analytical scope beyond infectious and survival outcomes, addressing a previously underexplored domain in chlorhexidine bathing research.

Given the uncertainty surrounding the effect on severity scores, it is important to examine whether this potential signal translates into measurable differences in objective clinical outcomes such as ICU length of stay. This meta-analysis demonstrated that patients undergoing bathing with 2% CHG had a mean ICU length of stay 0.07 days (approximately 1.7 h) longer than those receiving SAW, contradicting previous studies [14, 28] which did not demonstrate a statistically significant association between these variables.

Although the magnitude of this difference is clinically negligible, the direction of the effect contrasts with the initial hypothesis that reducing healthcare-associated infections through 2% CHG bathing would translate into shorter ICU stays. This discrepancy suggests that any potential impact of CHG on infectious outcomes may not be sufficient to meaningfully influence broader indicators such as length of stay, which are determined by multiple and interacting clinical factors.

The magnitude of the effect is extremely small and unlikely to be clinically meaningful at the individual patient level. Second, the pooled estimate was overwhelmingly driven by a single very large non-randomized study contributing more than 99% of the statistical weight. Such dominance raises concerns regarding aggregation bias and limits the generalizability of the summary effect.

Third, randomized cluster trials did not demonstrate a statistically significant difference in length of stay. This divergence between randomized and observational evidence suggests the possibility of residual confounding in non-randomized studies. For example, differences in discharge policies, bed management practices, and case-mix adjustments may have influenced observed length of stay independently of the bathing intervention.

Additionally, length of stay is a complex systems-level outcome affected by organizational, logistical, and structural factors beyond infection prevention measures. Therefore, causal attribution of a minimal length of stay difference to chlorhexidine bathing alone is methodologically tenuous.

A sensitivity analysis excluding studies classified as having a high risk of bias was also performed for the length of stay outcome (Supplementary Material, Fig. 6). After excluding these studies, no statistically significant difference was observed between groups (MD 0.04 [95% CI − 0.47; 0.56], *p* = 0.87; I² = 0%), indicating absence of heterogeneity.

Although statistical heterogeneity was absent, the wide confidence interval indicates substantial imprecision. In addition, the estimate remained largely informed by observational data. Therefore, the available evidence does not support a clinically meaningful or causally established effect of 2%CHG bathing on ICU length of stay.

### Limitations

It is important to acknowledge the limitations of this study. Notably, substantial heterogeneity was observed, particularly for mortality and clinical severity outcomes. Furthermore, the limited number of randomized controlled trials and the inability to perform meta-regression analyses restricted a more in-depth exploration of potential sources of variability.

Although formal assessment of publication bias through funnel plots was not conducted due to the small number of studies per outcome, the possibility of reporting or publication bias cannot be ruled out. Studies demonstrating reductions in healthcare-associated infections may be more likely to be published, whereas neutral or negative findings especially for non-infectious outcomes such as clinical severity or length of stay may remain unpublished.

In addition, several included studies were observational and single-center in design, increasing susceptibility to selective reporting. Therefore, the absence of formal funnel plot assessment should not be interpreted as evidence of absence of publication bias, and the certainty of evidence was interpreted with appropriate caution.

Finally, considerable clinical variability was observed among the included populations, encompassing medical, surgical, oncological, and immunosuppressed patients, which may have influenced the pooled estimates. Such variability limits direct comparability across studies and further reinforces the need for cautious interpretation. Nonetheless, as this review aimed to evaluate critically ill patients as a broad population, future analyses stratified by comorbidity profiles may help generate more precise estimates.

## Conclusion

Daily bathing with 2% chlorhexidine gluconate was not associated with a statistically significant reduction in the risk of death or clinical severity, with both outcomes supported by very low certainty of evidence. A small increase in ICU length of stay (0.07 days) was observed; however, the certainty of evidence was low and the magnitude of effect is unlikely to be clinically meaningful.

Beyond updating the quantitative estimates, the present review contributes materially to the current body of knowledge in several ways. First, by restricting inclusion to daily 2% chlorhexidine bathing compared exclusively with soap and water, we reduced intervention heterogeneity present in earlier syntheses and provided a more clinically homogeneous comparison framework. Second, we expanded the scope of evaluation beyond infectious outcomes and mortality by formally synthesizing clinical severity indices, an outcome domain not previously meta-analyzed in this context. Third, the inclusion of recently published large-scale studies increased statistical precision and allowed a more robust assessment of whether accumulating evidence modifies prior conclusions. Finally, by examining the differential influence of randomized and non-randomized evidence, our findings offer a more nuanced interpretation of the apparent effects on ICU length of stay. Collectively, these elements refine, rather than merely replicate, previous evidence syntheses.

## Supplementary Information

Below is the link to the electronic supplementary material.

Supplementary Material 1 (DOCX 623 KB)

Supplementary Material 2 (PDF 144 KB)

## Data Availability

No datasets were generated or analysed during the current study.
