# Supplementary material for: Effects of daily bathing with 2% chlorhexidine compared to bathing with soap and water on risk of death, clinical severity, and intensive care unit length of stay in critically ill patients: an updated systematic review and meta-analysis
Source: Eur J Clin Microbiol Infect Dis. 2026 Apr 18;45(8):2195–205. doi: 10.1007/s10096-026-05513-5 (PMC13428691; doi:10.1007/s10096-026-05513-5)
Supplement: Supplementary file 1 — Supplementary Material 1 (DOCX 623 KB) [file 10096_2026_5513_MOESM1_ESM.docx]

**SUPPLEMENTARY MATERIAL**

| **Study** | **Domain**  **1a** | **Domain**  **1b** | **Domain**  **2** | **Domain**  **3** | **Domain**  **4** | **Domain**  **5** | **Overall risk** |
| --- | --- | --- | --- | --- | --- | --- | --- |
| Bleasdale (2007) | Low | Low | Some concerns | Low | Some concerns | Low | Some concerns |
| Reis (2022) | Low | Low | Some concerns | Low | Low | Some concerns | Some concerns |
| Tomazini (2016) | Low | Low | Some concerns | Some concerns | Low | Low | Some concerns |

**Figure 1** Risk of bias summary for cluster-randomized trials (RoB 2)

Domain 1a: randomization process; Domain 1b: timing of identification and recruitment of individual participants in relation to timing of randomization; Domain 2: deviations from the intended interventions; Domain 3: missing outcome data; Domain 4: measurement of the outcome; Domain 5: selection of the reported result

**Figure 2** Risk of bias summary for non-randomized studies (ROBINS-I)

| **Study** | **Domain**  **1** | **Domain**  **2** | **Domain**  **3** | **Domain**  **4** | **Domain**  **5** | **Domain**  **6** | **Domain**  **7** | **Overall** |
| --- | --- | --- | --- | --- | --- | --- | --- | --- |
| Cassir (2015) | Moderate | Low | Low | Moderate | Low | Moderate | Low | Moderate |
| Chang (2025) | Serious | Moderate | Low | Moderate | Low | Moderate | Moderate | Serious |
| Duszyńska (2017) | Serious | Moderate | Low | Low | Moderate | Moderate | Low | Serious |
| Kengen (2018) | Moderate | Low | Low | Moderate | Low | Low | Low | Moderate |
| Lin (2025) | Moderate | Low | Low | Moderate | Low | Low | Moderate | Moderate |
| Suh (2021) | Serious | Moderate | Moderate | Moderate | Moderate | Low | Low | Serious |
| Tarakçıoğlu Çelik (2025) | Moderate | Low | Moderate | Moderate | Moderate | Low | Low | Moderate |

Domain 1: confounding; Domain 2: selection of participants; Domain 3: classification of interventions; Domain 4: deviations from intended interventions; Domain 5: missing data; Domain 6: measurement of outcomes; Domain 7: selection of the reported result

**Figure 3 -** Grading of Recommendations Assessment, Development, and Evaluation (GRADE)

| **Outcome** | **No. of studies** | **Study**  **design** | **Risk of bias** | **Inconsistency** | **Indirectness** | **Imprecision** | **Publication bias** | **Other considerations** | **Certainty of the evidence (GRADE)** |
| --- | --- | --- | --- | --- | --- | --- | --- | --- | --- |
| Death | 7 | CRTs + Observational | serious | not serious | serious | serious | undetected | none | ⨁◯◯◯ Very low |
| Clinical severity | 6 | CRTs + Observational | serious | not serious | serious | serious | undetected | none | ⨁◯◯◯ Very low |
| Length of stay | 7 | CRTs + Observational | serious | not serious | serious | serious | undetected | none | ⨁⨁◯◯ Low |

**
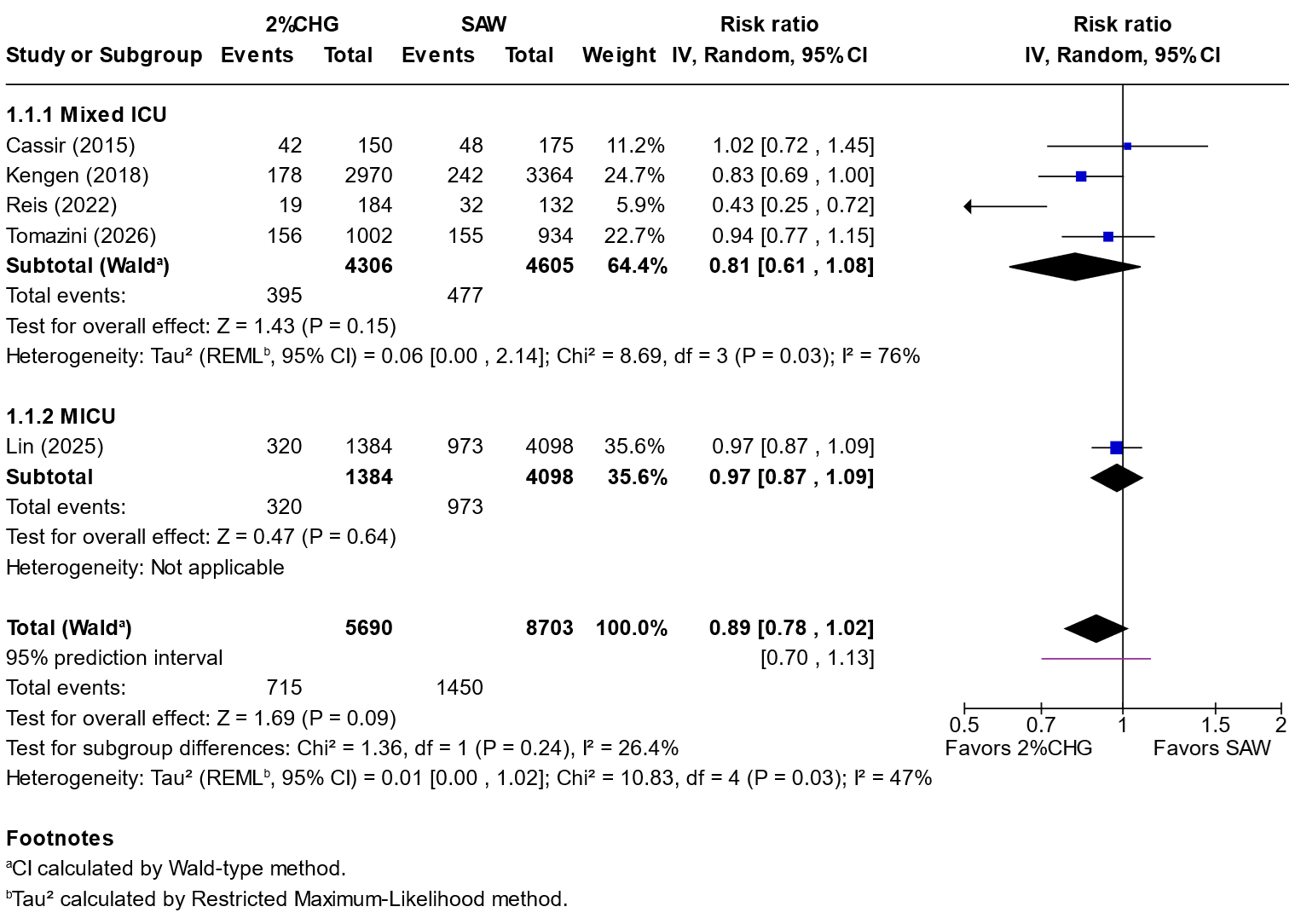
Figure 4** Deaths excluding all studies classified as having a high risk of bias

**
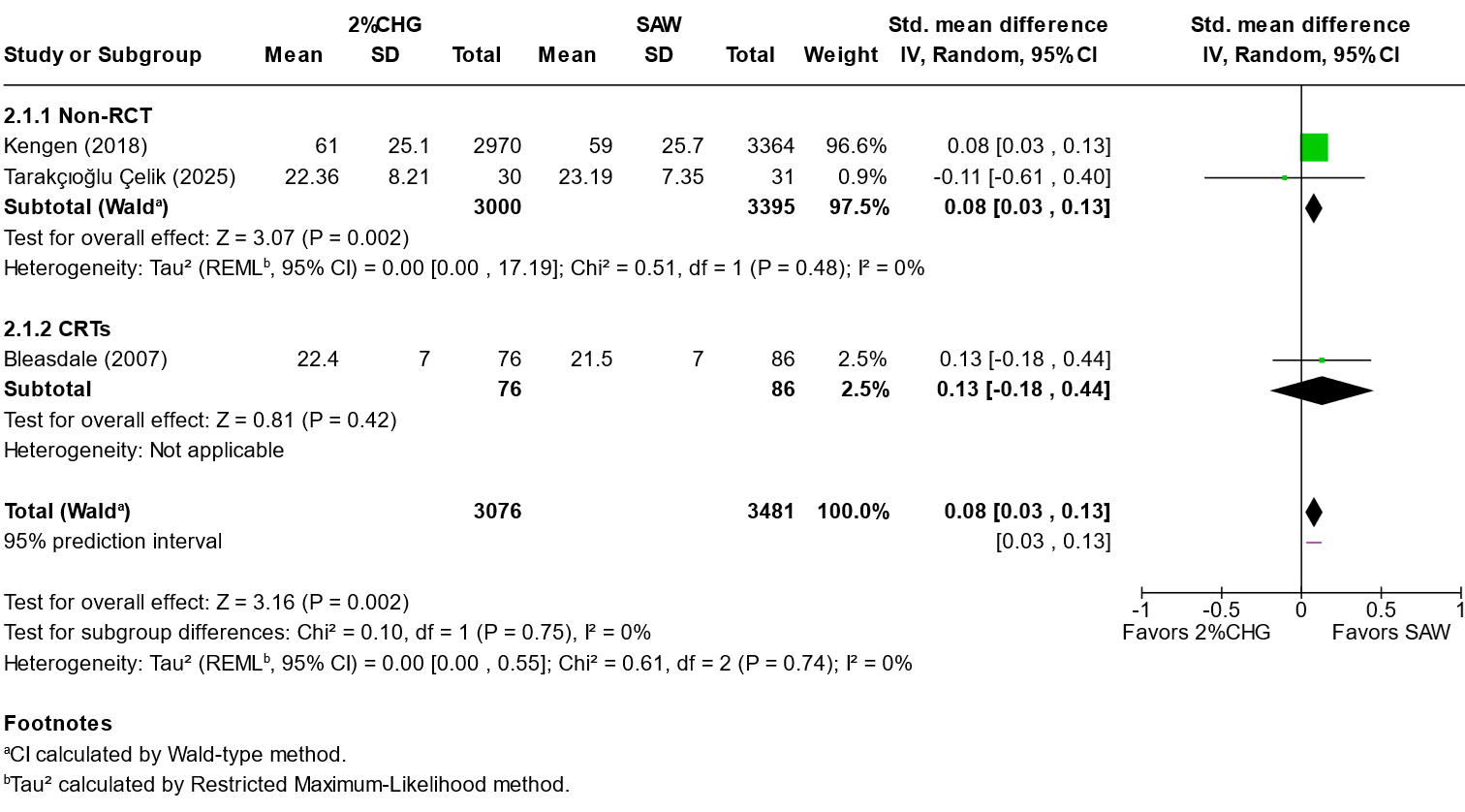
Figure 5** Clinical Severity excluding all studies classified as having a high risk of bias


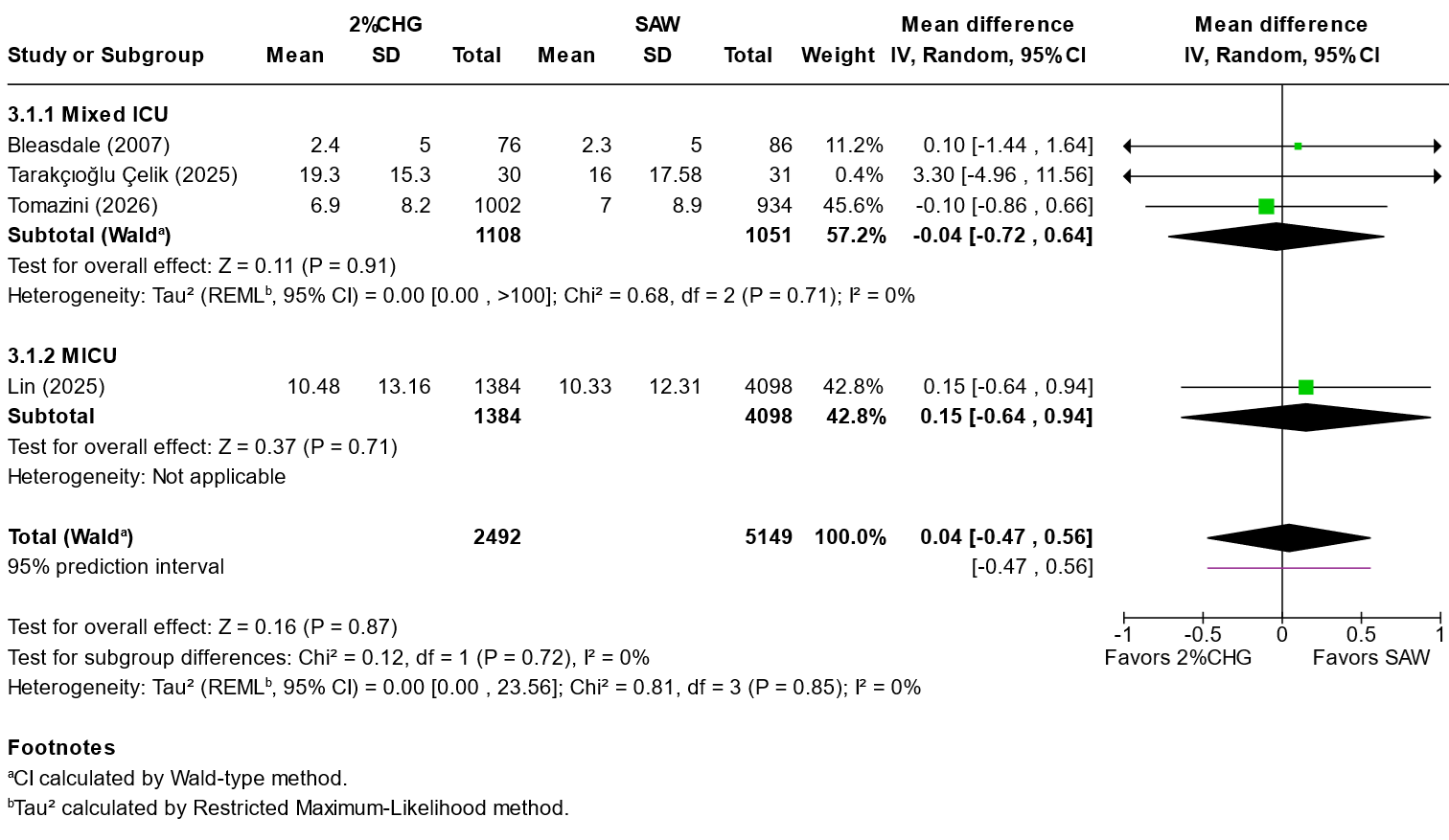
**Figure 6** Average length of stay in the ICU excluding all studies classified as having a high risk of bias

**Figure 7** Comparative table of the current meta-analysis with previous meta-analyses

| **Characteristic** | **Lewis (2019)** | **Peixoto (2024)** | **Present Study** |
| --- | --- | --- | --- |
| Primary focus | Hospital‐acquired infections | CLABSI prevention | Mortality, severity, LOS |
| CHG concentration | Variable | 2% (mainly wipes) | 2% only |
| Bathing frequency | Variable | Daily + alternate-day | Daily only |
| Comparator | Various (including usual care) | Not always SAW | Soap and water only |
| Study designs | RCTs | RCTs | CRTs + non-RCTs |
| Sample size | 24,472 | 20,188 | 33,860 |
| Mortality effect | No significant effect | No significant effect | Borderline, very low certainty |
| Clinical severity | Not evaluated | Not evaluated | Evaluated (high heterogeneity) |
| LOS | No significant reduction | No significant reduction | +0.07 days (clinically negligible) |
| New studies included | Until 2018 | Until 2022 | Until 2026 |
| Randomized studies not included in previous reviews | - | - | Tomazini (2026) |

Figure 8 Characteristics of intervention protocols and sources of clinical heterogeneity across included studies

| **Primary author (year)** | **Intervention** | **Application Method** | **Control** | **Bathing frequency** | **ICU type** | **Notes on potential heterogeneity** |
| --- | --- | --- | --- | --- | --- | --- |
| Bleasdale (2007) | 2% CHG-impregnated cloths (Sage) | 8 CHG cloths (neck to toes) + 2 non-medicated cloths for face; warmed before use | Soap and water (bar soap + 10 cloths) | Daily | 22-bed medical ICU (MICU) | Single-center; medical ICU only; crossover design; higher central line utilization in CHG arm |
| Cassir (2015) | 2% CHG impregnated cloths | 6 CHG cloths for body; 2 non-medicated for face; warmed before use | Soap and water (Phagoderm; Phagogène,  Nantes, France) | Daily | 14-bed medical ICU (MICU) | Retrospective analysis of prospective data; alternating 6-month periods; co-intervention with CHG dressings and oral CHG |
| Chang (2025) | 2% CHG-impregnated wipes | Standardized 8-step protocol (neck to abdomen, limbs, perineum, back, optional catheter cleansing) | Traditional liquid soap-water | Daily | 20-bed mixed medical and surgical ICU | Before–after design; implementation-level intervention; mixed ICU population; MDRO-focused outcomes |
| Duszyńska (2017) | 2% CHG impregnated cloths – universal decolonization | One package containing six  Cloths was used. One cloth was used for  a given body area and was disposed after a single use | Traditional soap-water bathing | Daily | 16-bed mixed ICU | Observational before–after design (three periods: pre, intervention, post); single-center study; temporal confounding possible; no randomization; potential influence of secular trends and infection control practices |
| Kengen (2018) | 2% CHG impregnated washcloths (Teleflex Medical Australia, Sydney, NSW) | Six cloths  was to be used to wash all body surfaces except for the face. All washcloths were discarded after use. | Soap and water | Daily | 31-bed mixed medical and surgical ICU | Retrospective interrupted time series (ITS) design; excluded patients with ICU stay < 24h. |
| Lin (2025) | 2% CHG bathing | Not fully detailed (routine clinical application in MICUs) | Soap and water | Daily and every-other-day (within-study comparison) | 3-bed medical ICU (MICU) | Observational design; variation in bathing frequency; inter-unit comparison; potential institutional confounding |
| Reis (2022) | 2% CHG detergent solution | Full-body bathing with 2% CHG detergent solution (no rinsing; air-dried; moisturizer applied after) | Neutral soap and water | Daily | 4-bed mixed ICUs (medical, neurosurgical, trauma/surgical, mixed) | Cluster randomized trial; multiple ICU types; baseline differences between groups (age, surgical profile); potential variability in adherence to bathing technique |
| Suh (2021) | 2% CHG | Six clean 2% CHG cloth wipes. Whole-body bathing (full or partial depending on patient condition) | Soap and water | Daily | 23-bed medical ICU (MICU) | Quasi-experimental (interrupted time series); incomplete adherence (~72.5%); high endemicity of VRE; presence of co-interventions (active surveillance, isolation measures) |
| Tarakçıoğlu Çelik (2025) | 2% CHG | 4% CHG, diluting it with water each time to obtain 2% CHG. (approximately 120 ml of 4% CHG solution were added to approximately 120 ml of water). | Standard soap and water | Daily | Oncology ICU (cancer patients) | Crossover design with only 78 patients; focused specifically on MRSA and VRE colonization. |
| Tomazini (2026) | 2% chlorhexidine digluconate | The bathing procedure consisted of applying chlorhexidine using soft polyester gloves, specifically designed for hospital bathing, ensuring that all applicable bathing surfaces (i.e., the entire body surface except for the eyes, inner ear, mouth, and areas with loss of skin continuity, such as open wounds and burnt regions) | Soap and water | Daily | 22-bed ICU | Large multicenter cluster-randomized crossover trial; focused on device-associated infections. |
