# Supplementary material for: Effects of daily bathing with 2% chlorhexidine compared to bathing with soap and water on risk of death, clinical severity, and intensive care unit length of stay in critically ill patients: an updated systematic review and meta-analysis
Source: Eur J Clin Microbiol Infect Dis. 2026 Apr 18;45(8):2195–205. doi: 10.1007/s10096-026-05513-5 (PMC13428691; doi:10.1007/s10096-026-05513-5)
Supplement: Supplementary file 2 — Supplementary Material 2 (PDF 144 KB) [file 10096_2026_5513_MOESM2_ESM.pdf]

SEARCH PROTOCOL	
PUBMED	**1. Infection**<br>(“critically ill” OR “critical illness” OR “intensive care units” OR hospital OR patients OR “intensive care” [MeSH]) AND (baths OR bath* [MeSH]) AND chlorhexidine AND (“hospital infection” OR “bloodstream infections” OR infection).<br><br>**2. Number of deaths**<br>(“critically ill” OR “critical illness” OR “intensive care units” OR hospital OR patients OR “intensive care” [MeSH]) AND (baths OR bath* [MeSH]) AND chlorhexidine AND (death*[MeSH] OR mortality OR “end of life”[MeSH).<br><br>**3. Clinical severity**<br>(“critically ill” OR “critical illness” OR “intensive care units” OR hospital OR patients OR “intensive care” [MeSH]) AND (baths OR bath* [MeSH]) AND chlorhexidine AND (“severity of illness index” OR “clinical severity”[MeSH]).<br><br>**4. Average length of stay**<br>(“critically ill” OR “critical illness” OR “intensive care units” OR hospital OR patients OR “intensive care” [MeSH]) AND (baths OR bath* [MeSH]) AND chlorhexidine AND (stay OR “length of stay”[MeSH] OR LOS).
SCOPUS	**1. Infection**<br>("critically ill" OR "critical illness" OR "intensive care units" OR hospital OR patients OR "intensive care" [MeSH]) AND (baths OR bath [MeSH]) AND chlorhexidine AND ("hospital infection" OR "bloodstream infections" OR infection).<br><br>**2. Number of deaths**<br>("critically ill" OR "critical illness" OR "intensive care units" OR hospital OR patients OR "intensive care" [MeSH]) AND (baths OR bath [MeSH]) AND chlorhexidine AND (death*[MeSH] OR mortality OR "end of life" [MeSH).<br><br>**3. Clinical severity**<br>("critically ill" OR "critical illness" OR "intensive care units" OR hospital OR patients OR "intensive care" [MeSH]) AND (baths OR bath [MeSH]) AND chlorhexidine AND (“severity of illness index” OR "clinical severity"[MeSH]).<br><br>**4. Average length of stay**<br>("critically ill" OR "critical illness" OR "intensive care units" OR hospital OR patients OR "intensive care" [MeSH]) AND (baths OR bath [MeSH]) AND chlorhexidine AND (stay OR "length of stay" [MeSH] OR LOS).
WEB OF SCIENCE	**1. Infection**<br>("critically ill" OR "critical illness" OR "intensive care units" OR hospital OR patients OR "intensive care" [MeSH]) AND (baths OR bath* [MeSH]) AND chlorhexidine AND ("hospital infection" OR "bloodstream infections" OR infection).<br><br>**2. Number of deaths**<br>(("critically ill" OR "critical illness" OR "intensive care units" OR hospital OR patients OR "intensive care" [MeSH]) AND (baths OR bath* [MeSH]) AND chlorhexidine AND (death*[MeSH] OR mortality OR "end of life" [MeSH)).<br><br>**3. Clinical severity**

	(("critically ill" OR "critical illness" OR "intensive care units" OR hospital OR patients OR "intensive care" [MeSH]) AND (baths OR bath* [MeSH]) AND chlorhexidine AND (“severity of illness index” OR "clinical severity"[MeSH])).<br><br>**4. Average length of stay**<br>(("critically ill" OR "critical illness" OR "intensive care units" OR hospital OR patients OR "intensive care" [MeSH]) AND (baths OR bath* [MeSH]) AND chlorhexidine AND (stay OR "length of stay"))
SCIENCE DIRECT	**1. Infection**<br>(("critically ill" OR "intensive care units" OR "intensive care" [MeSH]) AND (bath [MeSH]) AND chlorhexidine AND ("hospital infection" OR "bloodstream infections" OR infection)).<br><br>**2. Number of deaths**<br>(("critically ill" OR "intensive care units" OR "intensive care" [MeSH]) AND (bath [MeSH]) AND chlorhexidine AND (death OR mortality OR "end of life" [MeSH)).<br><br>**3. Clinical severity**<br>(("critically ill" OR "intensive care units" OR "intensive care" [MeSH]) AND (bath [MeSH]) AND chlorhexidine AND AND (“severity of illness index” OR "clinical severity"[MeSH])).<br><br>**4. Average length of stay**<br>(("critically ill" OR "intensive care units" OR "intensive care" [MeSH]) AND (bath [MeSH]) AND chlorhexidine AND (stay OR "length of stay"))
COCHRANE	**1. Infection**<br>(“critically ill” OR “critical illness” OR “intensive care units” OR hospital OR patients OR “intensive care” [MeSH]) AND (baths OR bath* [MeSH]) AND chlorhexidine AND (“hospital infection” OR “bloodstream infections” OR infection).<br><br>**2. Number of deaths**<br>(“critically ill” OR “critical illness” OR “intensive care units” OR hospital OR patients OR “intensive care” [MeSH]) AND (baths OR bath* [MeSH]) AND chlorhexidine AND (death*[MeSH] OR mortality OR “end of life”[MeSH).<br><br>**3. Clinical severity**<br>(“critically ill” OR “critical illness” OR “intensive care units” OR hospital OR patients OR “intensive care” [MeSH]) AND (baths OR bath* [MeSH]) AND chlorhexidine AND (“severity of illness index” OR “clinical severity”[MeSH]).<br><br>**4. Average length of stay**<br>(“critically ill” OR “critical illness” OR “intensive care units” OR hospital OR patients OR “intensive care” [MeSH]) AND (baths OR bath* [MeSH]) AND chlorhexidine AND (stay OR “length of stay”[MeSH] OR LOS).
EMBASE	**1. Infection**<br>('critically ill' OR 'critical illness' OR 'intensive care units' OR 'hospital' OR 'patients' OR 'intensive care'/exp) AND ('baths' OR 'bath*'/exp) AND 'chlorhexidine' AND ('hospital infection' OR 'bloodstream infections' OR 'infection').

	**2. Number of deaths**<br>('critically ill' OR 'critical illness' OR 'intensive care units' OR 'hospital' OR 'patients' OR 'intensive care'/exp) AND ('baths' OR 'bath*'/exp) AND 'chlorhexidine' AND ('death*'/exp OR 'mortality' OR 'end of life'/exp).<br><br>**3. Clinical severity**<br>('critically ill' OR 'critical illness' OR 'intensive care units' OR 'hospital' OR 'patients' OR 'intensive care'/exp) AND ('baths' OR 'bath*'/exp) AND 'chlorhexidine' AND ('severity of illness index' OR 'clinical severity'/exp).<br><br>**4. Average length of stay**<br>('critically ill' OR 'critical illness' OR 'intensive care units' OR 'hospital' OR 'patients' OR 'intensive care'/exp) AND ('baths' OR 'bath*'/exp) AND 'chlorhexidine' AND ('stay' OR 'length of stay'/exp OR 'los').
